# Evidence-Based Policymaking in Times of Acute Crisis: Comparing the Use of Scientific Knowledge in Germany, Switzerland, and Italy

**DOI:** 10.1007/s11615-022-00382-x

**Published:** 2022-04-04

**Authors:** Susanne Hadorn, Fritz Sager, Céline Mavrot, Anna Malandrino, Jörn Ege

**Affiliations:** 1 KPM Center for Public Management, University of Bern, Bern, Switzerland; 2 Institute of Social Sciences, University of Lausanne, Lausanne, Switzerland; 3 Department of Political and Social Sciences, University of Bologna, Bologna, Italy; 4 University of Konstanz, Konstanz, Germany

**Keywords:** Evidence-based policies, Policy advice, Expert system, Scientific knowledge, Crisis management, COVID-19, Evidenzbasierte Politik, Politikberatung, Expertensystem, Wissenschaftliche Evidenz, Krisenmanagement, COVID-19

## Abstract

This article studies how different systems of policy advice are suited to provide relevant knowledge in times of acute crisis. The notion of evidence-based policymaking (EBP) originated in the successful 1997 New Labour program in the United Kingdom to formulate policy based not on ideology but on sound empirical evidence. We provide a brief overview of the history of the concept and the current debates around it. We then outline the main characteristics of the policy advisory systems in Germany, Switzerland, and Italy through which scientific knowledge—in the form of either person-bound expertise or evidence generated through standard scientific processes—was fed into policy formulation processes before the COVID-19 crisis. Whereas EBP takes place in the form of institutionalized advisory bodies and draws on expertise rather than on evidence in Germany, the system in Switzerland focuses more on the use of evidence provided through external mandates. Italy has a hybrid politicized expert system. The article then analyzes how this different prioritization of expertise vs. evidence in the three countries affects policymakers’ capacity to include scientific knowledge in policy decisions in times of acute crisis. The comparison of the three countries implies that countries with policy advisory systems designed to use expertise are better placed to incorporate scientific knowledge into their decisions in times of acute crisis than are countries with policy advisory systems that relied primarily on evidence before the COVID-19 crisis.

**Supplementary Information:**

The online version of this article (10.1007/s11615-022-00382-x) contains supplementary material, which is available to authorized users.

## Abstract (de)

Dieser Artikel untersucht die Eignung unterschiedlicher Politikberatungssysteme im Hinblick auf die Bereitstellung von relevantem Wissen für politische Entscheidungen in Krisenzeiten. Das Konzept der evidenzbasierten Politikgestaltung (EBP) geht auf das erfolgreiche New-Labour-Programm aus dem Jahr 1997 im Vereinigten Königreich zurück, welches propagierte, dass Politik nicht auf der Grundlage von Ideologie, sondern basierend auf soliden empirischen Erkenntnissen formuliert werden soll. Der Artikel gibt zuerst einen kurzen Überblick über die Geschichte des Konzepts der EBP und die aktuellen Debatten darüber. Anschließend werden die Hauptmerkmale der Politikberatungssysteme in Deutschland, der Schweiz und Italien skizziert, durch die traditionell wissenschaftliche Erkenntnisse in die jeweiligen Politikprozesse einfließen. Dabei wird zwischen personengebundener Expertise und Evidenz aus wissenschaftlichen Analysen unterschieden. Während in Deutschland evidenzbasierte Politikgestaltung in Form von institutionalisierten Beratungsgremien stattfindet, die sich eher auf Expertise stützt, setzt die Schweiz eher auf die Nutzung von Evidenz, die durch externe Mandate bereitgestellt wird. Italien besitzt ein hybrides politisiertes Expertensystem. Der Artikel analysiert, wie sich diese unterschiedliche Priorisierung von Expertise gegenüber Evidenz in den drei Ländern auf die Fähigkeit der politischen Entscheidungsträger*innen auswirkt, in Krisenzeiten wissenschaftliche Erkenntnisse in politische Entscheidungen einzubeziehen. Der Vergleich der drei Länder zeigt, dass Länder mit Politikberatungssystemen, die auf die Nutzung von Expertise ausgerichtet sind, besser in der Lage sind, in Krisenzeiten wissenschaftliche Erkenntnisse in ihre Entscheidungen einfließen zu lassen, als Länder, die sich traditionell in erster Linie auf Evidenz stützen.

## Introduction

This article studies how different systems of policy advice are suited to provide relevant knowledge in times of acute crisis. The notion of evidence-based policymaking (EBP) became popular in the 1990s, particularly with the Labour government under Tony Blair in the United Kingdom (Frey and Ledermann 2010) and its famous slogan “What matters is what works” (Nutley et al. 2007). Evidence-based policymaking “relies on the assumption that we can make policies work better if we understand how policy mechanisms bring about change in social systems to achieve desired outcomes” (Sanderson 2002, p. 2). Policy decisions are expected to be the result of a thorough and rigorous consideration of scientific evidence (Parkhurst 2017). Contrary to this optimistic view, critics of EBP point out that public policymaking is an inherently political matter in which the main objective for different stakeholders is to promote their interests in the best possible way (Parkhurst 2017). In practice, this means that decisions in favor of or against certain policies are driven not by scientific evidence but by ideology.

Still, many countries have established structures and processes with the aim to promote the use of scientific information in policy formulation. This paper addresses the question of how scientific knowledge is fed into the policy process in Germany, Switzerland, and Italy (through which structures and processes) in noncrisis times and what form that scientific knowledge takes. We then analyze how policy advisory systems operate under situations of “creeping crises” (Boin et al. 2020; ’t Hart and Boin 2001), such as the COVID-19 pandemic. In this crisis type, policymakers are confronted with the central challenge of translating “ambiguous information to a strategic decision making agenda” (Boin et al. 2020, p. 133).

Policy formulation in Germany has a long tradition of obtaining policy-relevant knowledge through different types of advisory bodies, often in the form of expertise (Veit et al. 2017, p. 90). Italy has a system of politicized, primarily internal expertise (Capano 2020; Galanti and Saracino 2021). In contrast, in Switzerland, scientific advice mostly takes the form of short-term mandates given to external advisors, who file written reports containing evidence (Himmelsbach 2019). Based on such a “diverse case” selection (Seawright and Gerring 2008, p. 300), we tested whether these systemic differences had an impact on policymakers’ ability to incorporate rapidly needed scientific knowledge into policy decisions during the COVID-19 crisis. Comparing the contrasting policy advisory systems increased the representativeness of our sample and facilitated careful generalizations.

Before analyzing the use of scientific knowledge during the COVID-19 pandemic, we first examine the structures and processes through which scientific knowledge was fed into the political processes in the three countries before the COVID-19 crisis.

## What does EBP look like in Germany, Switzerland, and Italy?

The underlying assumption of EBP is that policymakers can design improved policies when “existing knowledge on the effectiveness and efficiency of interventions is systematically reviewed” (Balthasar 2010, p. 333). In this paper, we focus on this praxis-based notion because politicians relied heavily on the promises of EBP to help tackle the pandemic. However, as Head (2010, p. 80) has argued, “the early hopes of large and rapid improvements in policies and programs […] have not materialized as readily as anticipated.” First, certain scholars criticize the proponents of the EBP approach for not paying sufficient attention to the political realities of the policymaking process (Newman 2017; Parkhurst 2017), where scientific knowledge is not the only basis for decision-making and, above all, is often not the most important. Under the label “science-policy nexus,” Robert Hoppe (2005) actually shows that there is a wide array of possible nuances in the arrangements between scientists and politicians, ranging from genuine learning to pure knowledge use, depending on the specific circumstances. Second, a major problem of EBP lies in the different motivations and goals that drive researchers and policymakers. While researchers do not primarily strive at informing policy decisions but rather are seeking to publish their results in scientific outlets (which can take a long time), policymakers need timely and relevant information depending on the deadlines set by the policy processes (Sager et al. 2020). These two issues are thus major obstacles to EBP (Cairney 2016; Stoker and Evans 2016). The appropriate limits of the expert’s role in policymaking have also been widely discussed: Scientists can see their roles as merely informative without regard to the final policy decision, explicitly engage in issue-advocating, or find a balance in the role of “honest broker” by providing politicians with various policy alternatives (Pielke 2007). Another body of literature is specifically aimed at examining the practical features of “scientific advisory committees,” such as their size, composition, cohesion, or procedures, to determine their optimal contribution to policymaking (Behdinan et al. 2018). In any case, it is important to acknowledge the interdependency between experts and decision-makers in many governance areas and to consider science and policy as constantly co-evolving social processes that intersect in “science–policy interfaces” where joint knowledge is produced (van den Hove 2007). In this paper, we examine institutional policy advisory system performance in times of crisis to help solve these problems and to better integrate evidence into policymaking (Newman 2017).

### Different Designs of Policy Advisory Systems

In the analysis of “politically relevant knowledge” provided through policy advisory systems, Ledermann (2014, p. 453) differentiates between *expertise *and *evidence*. Expertise captures knowledge that is person-bound, meaning that individuals provide policymakers with their professional expertise acquired through their long-term and in-depth involvement with a topic. Expertise is integrated in the political process, e.g., by political advisory bodies that consult the responsible public administration and policymakers. In contrast, evidence is “independently observable and verifiable” and generated by applying scientific standards (i.e., through systematic and intersubjectively traceable analyses) (Ledermann 2014, p. 456). Evidence is thus explicitly not person-bound. Furthermore, the form of transmission also differs: Evidence must take written form and be accessible on paper or electronically, whereas expertise can also be transmitted verbally (Ledermann 2014). Expertise and evidence are the two ends of a continuum of many mixed forms in which scientific advice is provided. There are various factors that impact the form of knowledge transfer. For example, Leifeld and Schneider (2012) distinguish different kinds of information, such as political and technical information exchange. In the following, we employ the evidence–expertise dichotomy without claiming that it does full justice to the social complexity of knowledge transfer. However, it offers a general framework for comparison that allows us to place national systems within the range of mixed forms and show whether a certain policy advice system leans more toward one or the other type.

### The Use of Scientific Knowledge in Germany: A Focus on Expertise

The institutionalization of knowledge transfer in Germany is characterized by a “long-standing tradition for the establishment of advisory bodies to the government with some of today’s advisory bodies established shortly after the end of WWII” (Veit et al. 2017, p. 90). The clarity of the diverse advisory bodies’ missions, the addressees of the advice they provide (single vs. several ministries), their resources and institutional setting, and the degree to which their recommendations are binding on the addressees vary (Weingart and Lentsch 2008). While some are composed purely of scientists, other advisory bodies additionally include representatives from different ministries and/or interest groups. Overall, however, these bodies are similar in the sense that they are close to the government but are still independent, and their overall goal is to deliver advice to ministries (Veit et al. 2017). Hence, they provide ministries with current scientific knowledge and thereby compensate for potentially lacking expertise and insufficient administrative capacities. Fleischer (2015, p. 318) argues that “in this way, advisory bodies contribute to the output legitimacy of executive decision-making by using their expertise and reputation to safeguard the common good.”

Looking at another source of scientific knowledge, namely evidence provided through policy evaluations by external advisors, the picture in Germany is different. Although the importance of evaluations has increased, there is still great potential for further institutionalization. For instance, there exists neither an evaluation legislation nor evaluation policy at the national or state level, and the federal parliament is not equipped with an autonomous evaluation entity (Stockmann and Meyer 2020, p. 171). Overall, Stockmann and Meyer (2020) argue that there still exists a rather undeveloped evaluation culture in Germany.

### The Use of Scientific Knowledge in Switzerland: External Mandates as Key Providers of Evidence

In Switzerland, most of the public administration’s needs for policy advice are covered by short-term mandates with external contractors. They include policy evaluations as well as mandates “to clarify the economic and ecological effects of a policy alternative, to explore its legal scope for action or to obtain proposals for the optimal design of an instrument” (Himmelsbach 2019, p. 465). The written reports, which are the product of such mandates, are produced by private consultancies or universities. Unlike in Germany, the institutionalization of this consulting activity is particularly strong in the area of policy evaluations. Since 2000, the Federal Constitution has contained an article that requires from parliament that public policies be assessed for their effectiveness (Widmer 2017). Additionally, the federal parliament has its own internal evaluation unit called Parliamentary Control of the Administration (Swiss Parliament 2021). As concerns the use of evaluations through the public administration, “there is occasional systematic use of evaluation in policy formulation” whereby evaluations are used more commonly in a symbolic way in order to “increase the attractiveness of legislative bills” (Widmer 2020, p. 214).

In terms of internal advisors, Himmelsbach (2019, p. 459) argues that in Switzerland, “unlike in countries such as Italy, Germany or the UK, there has been very limited development of specialized research and advisory departments within the administration.” One of the instruments that we characterize as bodies internal to the administration, since they belong to the “decentralized federal administration” (Federal Chancellery 2021), are the extraparliamentary commissions. They are composed of scientists; top officials from the public administration, including representatives of lower tiers of government (i.e., cantons and municipalities); and associations. According to Himmelsbach (2019, p. 462), these commissions “primarily draw on the experience of their members when preparing reports. External analyses or in-depth investigations by commission members are rare.” Hence, such commissions primarily provide expertise rather than evidence. However, their importance has gradually decreased over time, both numerically and in terms of their inclusion in policy formulation (Himmelsbach 2014, 2019).

In sum, policy advice in Switzerland stems largely from the external advisors through short-term policy evaluation mandates in the form of reports rather than through advisory bodies. Thus, policy-relevant knowledge is mainly obtained in the form of evidence rather than by drawing on person-bound expertise.

### The Use of Scientific Knowledge in Italy: Internal Expertise Between Politicization and a Legalistic Culture

Policy advice practices present a hybrid nature in Italy (Galanti and Lippi 2019), within a policy advisory system that is still undeveloped and politicized (Capano 2020). The Napoleonic administrative tradition is combined with great variety in the diffusion of policy knowledge at both the national (ministerial) and subnational level (Dente 1997), while the politicization of policy advice emerges as a specific type of patronage (Di Mascio and Natalini 2016). The problematic nature of patronage resides in the senior civil servants’ subordination to politicians and the consequent danger of ending up with a less autonomous and professionalized civil service not capable of doing policy work that transcends the legal domain (Di Mascio and Natalini 2013; Galanti and Lippi 2019; Gouglas et al. 2017). The prevalence of legalistic knowledge in the administration makes Italy an inhospitable system for EBP, which has therefore a minor role therein (Capano 2020; Galanti and Saracino 2021).

In Italy, one of the main carriers of knowledge involvement in policymaking is represented by regulatory impact assessments, which were introduced experimentally in 1999^1^ and are emblematic of formalized procedures that embed policy evaluation directly in lawmaking processes (Melloni 2020). However, unlike in Switzerland, the use of policy evaluations remains limited in Italy because of the timing of evaluations, the lack of funding, and the difficulty of including evaluation results in the policy cycle at the right time (Melloni 2020). Based on these characteristics of the advisory system, Italian policymakers are more likely to rely heavily on fast, person-bound expertise rather than on evidence generated through scientific procedures (Ledermann 2014). This assumption is supported by the fact that even in nonemergency times, Italy adopts an emergency style of policymaking (Dente and Regonini 1989) in which problem pressure (Galanti and Lippi 2019) makes policymakers more likely to rely on faster expertise than on evidence, for which the production process can be more time-demanding. In fact, most policy advice is institutionalized inside ministerial cabinets (Galanti and Saracino 2021).

## How Do the Different EBP Systems Meet Political Needs in Times of Acute Crisis? An Analytical Model

We now present an analytical model to investigate the fit between different policy advisory systems and the requirement to quickly include newly emerging scientific knowledge into policymaking processes in times of crisis. The model proposes that depending on how policy advisory systems in noncrisis times prioritize either the use of evidence or expertise, different “logics of scientific knowledge” are preferred. The three logics of scientific knowledge are salience, credibility, and representativeness (Cash et al. 2003; cited in Veit et al. 2017). According to Veit et al. (2017, p. 87), “salience denotes the relevance and timeliness of advisory knowledge for policy-makers, […] credibility refers to whether the production of knowledge follows established epistemological standards, i.e. whether it is scientifically robust [, and] representativeness denotes whether knowledge is produced in an unbiased way by considering all relevant interests.” We argue that outside the COVID-19 crisis, these three logics receive different degrees of importance depending on the type of policy advisory system anchored in each country and the associated knowledge used (evidence vs. expertise). In times of crisis, the prioritization of these three logics might change, with salience becoming the most important logic. This is because politicians require immediate advice to make timely decisions responding to the crisis.

We argue that policy advisory systems working on the basis of expertise, which scores high on salience, are better prepared to absorb scientific knowledge in times of crisis. This is because systems familiar with the use of expertise increase the ability of policymakers to feed the urgently needed and rapidly generated scientific knowledge into policy formulation as compared with systems normally prioritizing evidence.

### Different Prioritization of the Three Logics of Scientific Knowledge

In this section, we examine the pertinence of the three logics of scientific knowledge with respect to expertise and evidence.

#### Expertise

As defined by Ledermann (2014), expertise is person-bound knowledge provided by experts. Expertise can be fed into policymaking through interdisciplinary advisory bodies, for example. The experts’ knowledge is rooted in long-term involvement in each policy area and not generated through systematic processes following scientific standards (Ledermann 2014). It can therefore be provided in real time, often in oral form, and is thus a flexible and immediate source of scientific knowledge for policymakers. We formulate the following expectations regarding the prevalence of salience, credibility, and representativeness in expertise-based systems:

- **Salience: **Compared with evidence, expertise has the advantage of a situation-specific applicability (Ledermann 2014). The logic of salience is thus strongly represented by expertise. This is due to the practical proximity of advisory bodies to policymakers that allows them to base their advice on policymakers’ needs and to provide knowledge in a timely manner (see Weingart and Lentsch 2008).
- **Credibility:** Expertise in the form of personalized and ad hoc advice provided by advisory boards is primarily based on the experts’ professional experience (Ledermann 2014). In these cases, the production of knowledge does not follow standard scientific processes. Hence, credibility (understood as strictly following scientific standards when generating knowledge) is not a priority.
- **Representativeness: **The degree of representativeness of expertise depends strongly on the selection of the experts. If an advisory group consists of only one professional group (e.g., economists), and experts from other disciplines (e.g., sociologists) are not represented, it can be assumed that representativeness is restricted. If there is a balance of different disciplines, however, representativeness can be high.

#### Evidence

Evidence is research-based information that has been generated through processes applying scientific standards (Ledermann 2014). In addition, evidence must be recorded in written form and is explicitly not person-bound (in fact, the sender is often deliberately not communicated) (Ledermann 2014). Based on these features of evidence, the following expectations are formulated:

- **Salience: **Salience is expected to be less strongly emphasized by evidence because generating knowledge through processes following scientific standards can take a long time. This delays the provision of evidence (e.g., provided through policy evaluations), which may come too late to satisfy the need for policymakers’ advice (Sager et al. 2021).
- **Credibility:** The credibility of the evidence can be considered high, since one of the basic requirements of evidence is that it be generated through scientifically sound procedures (Ledermann 2014). For instance, evaluations must meet established scientific standards set by national evaluation societies and thus generate objective and reproducible evidence.
- **Representativeness: **The representativeness of evidence obtained through policy evaluations is expected to be relatively high. This is because one principle of policy evaluations is the balanced weighting of different perspectives from different stakeholders. Thus, the study of a certain subject is based on a collection of different points of view, which are then evaluated using scientific methods.

In summary, expertise focuses on the logic of salience, while credibility is less pronounced, and the representativeness of expertise depends on the selection of experts. In contrast, evidence has a strong focus on credibility and on representativeness, while the logic of salience often cannot be prioritized (see Table 1).

**Table 1:** Contrasting logics of scientific knowledge for different types of knowledge and in times of crisis

*Type of scientific knowledge*	*Logic of scientific knowledge*		
	Salience	Credibility	Representativeness
Expertise	High	Low	Low–high
Evidence	Low	High	High
Demand in times of crisis	High	Low–moderate	Low–moderate

### Shifting Prioritization of the Three Logics of Scientific Knowledge in Times of Crisis

We argue that the needs of policymakers in times of crisis are special in terms of prioritizing the three logics of scientific knowledge. At the same time, the necessary scientific knowledge is often not yet available or at least not entirely certain. This is why the conditions under which politicians have to make decisions in times of acute crisis are very particular.

Parviainen et al. (2021, p. 236) summarize decision-making during the COVID-19 crisis “using Jerome R. Ravetz’s words: ‘facts are uncertain, values in dispute, stakes high and decisions urgent’ (Ravetz 1999, p. 649)”. If we apply this situation to the prioritization of the three logics of scientific knowledge, the following picture emerges:

- **Salience:** In the acute phase of a crisis, policymakers need to react rapidly because of the strong problem pressure. Accordingly, they prioritize salience, i.e., the provision of timely and relevant scientific knowledge.
- **Credibility:** Because of the short time policymakers have to react, the existing knowledge is often not fully secured (e.g., studies may have not yet gone through peer review processes). Accordingly, the logic of the credibility of scientific knowledge tends to be low or moderate in times of acute crisis.
- **Representativeness**: The generation of unbiased scientific knowledge that includes all relevant interests is also limited by the short-term nature of the decisions for which the knowledge is to be used. A targeted consideration of different perspectives is often possible only in later phases of crisis management, which is why representativeness is rated as low to moderate.

To sum up, Table 1 shows that the needs regarding the prioritization of the three logics during a crisis situation are better met by the characteristics of expertise than by those of evidence.

Table 1 shows that there is a large correspondence between the weighting of the three logics in the case of expertise and the demands on scientific knowledge in times of crisis. Thus, with a strong focus on salience, expertise seems to be the appropriate means to provide rapid knowledge to the policy formulation process in times of crisis. In contrast, the scientific processes needed to generate evidence are often too slow to provide relevant knowledge quickly enough. We therefore argue that countries with policy advisory systems that are designed to use expertise are better placed to incorporate scientific knowledge into their decisions in times of crisis than those countries with policy advisory systems that rely primarily on evidence.

## Research Design

The analysis builds on a comparative case study design by contrasting Germany, Switzerland, and Italy. The case selection was motivated by the very different institutionalization of the use of scientific knowledge before the pandemic. As outlined before, scientific knowledge is primarily used in the form of expertise by commissions or cabinets in the policy formulation process in Germany and Italy, whereas this happens mainly through evidence provided by short mandates in Switzerland. Moreover, while having different policy advisory systems, these countries were exposed to the same context conditions during the pandemic. This is the case regarding access to international expert recommendations such as those of the World Health Organization or the possibilities of cross-country policy learning given the rapid spread of the COVID-19. They are therefore considered as independent cases, and the analysis can focus on the way each country’s expert advisory system mediates the available evidence. Comparing these contrasting policy advisory systems constitutes a “diverse case” selection strategy (Seawright and Gerring 2008, p. 300), which intends to represent the full range of advisory systems. This strategy increases representativeness of cases, facilitates (careful) generalizations, and allows us to draw conclusions about how, in times of crisis, they may respond and absorb (sparse and often provisional) scientific knowledge differently. We analyzed the second wave of the COVID-19 crisis for two reasons: First, at the time of analysis, this was the most recent wave, and thus the most current data were available. Second, we chose to analyze the second wave rather than the first because, unlike at the beginning of the crisis, policy actors and advisors had already developed a routine in their exchanges. Therefore, this period of study is best suited to make meaningful analyses of how scientific knowledge was used during the pandemic. The exact dates of the second wave, however, differ for each country. We carried out our analysis based on a triangulation between three types of sources: media sources (newspapers, video declarations, news broadcasts), administrative and policy documents, and policy advice records. In particular, the media analysis concerned two major national newspapers for each country as well as additional press releases and video material, and we complemented it with written documents regarding the policies adopted and the policy advice provided by the examined committees. More details about our sources are available in Table A1 in the online annex. We use abbreviations for the sources in the footnotes; a full list can be found in the online annex. In order to empirically assess the salience, credibility, and representativeness of each national model, we used specific indicators in the form of questions for each country (see Table A2 in the online annex).

## Results

In the following sections, we analyze the influence of the differences in the above outlined policy advisory systems of the three countries on the ability of policymakers to absorb scientific knowledge during the COVID-19 pandemic. The comparison in the following section will allow us to detect patterns of change of individual systems in times of crisis compared with the situation before the crisis, which allows us to identify differences in how different policy advice systems cope with the same policy problem.

### Germany

In Germany, the second wave took place between early October 2020 and mid-January 2021, with an exponential growth of cases in October and another growth before Christmas (Schuppert et al. 2021). During this time, the German Länder and the federal government introduced several measures: While mask requirements and regulations on travel and gatherings were still in place, a partial lockdown started on November 2, 2020^2^, and was extended to school and retail shop closures on December 16, 2020 (Schuppert et al. 2021, p. 2; Naumann et al. 2020). Overall, introducing and justifying strict measures were more difficult given the successful management of the first wave. The German public and policymakers lost “the fear surrounding the pandemic to the extent that the narrative quickly took hold that it was less dangerous and contagious than health experts stated.”^3^

In 2001, the federal government adopted the Infection Protection Act. This law allows the federal government to formulate recommendations but leaves the execution to the Länder, which then decide on specific measures as well as their strictness, timing, and scope (Hegele und Schnabel 2021). Thus, decisions during the pandemic were primarily made jointly by the federal government and the Länder governments. The respective meetings served as coordination fora, and the decisions taken provided authoritative guidance to the Länder. The core policy decisions concerning the second wave were taken during three joint meetings:

- October 28, 2020: soft lockdown from November 2
- December 13, 2020: hard lockdown from December 16
- January 5, 2021: extension of hard lockdown until January 31

These decisions serve as a basis to assess the specific nature of EBP during the second wave (expertise vs. evidence). In Germany, the way in which scientific knowledge was used to inform decision-making at the joint meetings is characterized by *high salience, low (to medium) credibility*, and *low representativeness*.

#### High Salience

From the beginning of the pandemic, the federal government could quickly rely on scientific input from a variety of its own internal-to-government advisors. These included the standing commission on vaccinations, the national ethics council, and the national academy of science (Leopoldina), as well as its own scientific institutes—most importantly, the Robert Koch Institute and the Paul Ehrlich Institute. The heads of these institutes played a central role in communicating developments to the public and suggesting measures to fight the virus, often in the form of direct policy advice. In particular, eight individual experts, who are the heads of the most important scientific institutes and leading university researchers, can be considered close to policymakers.^4^ Chancellor Merkel and the heads of states would consult with them regularly to get their input, especially before important meetings.^5^

#### Low to Medium Credibility

Although expertise was generally produced in compliance with scientific standards, the high complexity and uncertainty that characterized the fight against COVID-19 required personal assessments of the experts consulted—for instance, if evidence was not in line with more general experiences or if evidence was itself contradictory. For example, policymakers followed the expertise of virologist Christian Drosten, physicist Viola Priesemann, and others to keep schools and nurseries closed after the January 5 meeting. This decision was taken even though the head of the University Hospital Dresden (Reinhard Berner) referred to a study by his institute that concluded that schools could be reopened.^6^ What increases the credibility of scientific knowledge in the German case is that almost all of the consulted experts are leading scientists in their fields.

#### Low Representativeness

German policymakers could rely on the scientific advice of research institutes and the national academy of science, which cover a variety of academic disciplines but have a clear focus on the natural sciences.^7^ A look at the group of experts who were consulted before the joint meetings confirms this impression. Four experts have a medical background (in particular, virology and epidemiology), two are physicists, and one works in bioinformatics, whereas only one member covers the social–psychological dimension of the pandemic.^8^ This led to substantial criticisms of the representativeness of EBP from other scientists,^9^ from different opposition parties (e.g., requesting an interdisciplinary scientific pandemic council)^10^, and from the public, resulting in a highly politicized debate. During demonstrations and gatherings of a heterogenous group called “Querdenker,” Wieler and Drosten especially became prime subjects of hate of anti–COVID-19 restriction protesters.^11^ While most leading scholars supported the government’s COVID-19 strategy in principle, some experts who were not part of the advisory system criticized certain measures^12^ or even rejected the danger of COVID-19 more generally.^13^

In sum, the high salience, the low to medium credibility, and the low representativeness in which scientific knowledge has been produced in the German case indicates that it can be best described as an expertise-based system.

To examine whether this expertise was actually considered by policymakers, we tracked the core policy decisions made during the three major government meetings and show how they align with expert recommendations. Table A3 in the online annex details the most prominent expert recommendations and compares them with the core decisions made at the three conferences.^14^ For instance, Christian Drosten, one of the most prominent experts, was in favor of an early but soft lockdown, with schools and stores to remain open. With the introduction of a soft lockdown at the October 28 meeting, this advice was followed. However, his advice for increased efforts in the tracing of outbreak clusters was not mentioned in the government decision. Nevertheless, we can conclude that Drosten’s advice was mostly followed. Furthermore, in Merkel’s justification of the decision for a hard lockdown during a debate in parliament, she made an explicit reference to the recommendations made in the sixth statement of the national science academy Leopolina.^15^ Overall, the decisions made at the meetings broadly followed expert advice even though some experts in some instances recommended stricter measures. One can also assume that the most important experts had direct access to policymakers. Merkel herself was usually on the side of the experts and in favor of stricter measures, but the necessity to get agreement from Länder governments allowed for less far-reaching and sometimes delayed measures. The “prevention paradox” experienced during the first wave made it also more difficult to justify and quickly introduce strict and comprehensive measures.^16^ In sum, we can conclude that policymakers did not deviate substantially from what (the majority of) experts recommended and that the German expertise-based system of EBP was able to effectively insert scientific knowledge into governmental decision-making.

### Switzerland

In Switzerland, the second wave lasted from mid-October 2020 to mid-January 2021 (BAG 2021b). After the nationwide restrictions of the first wave during spring 2020 (semi-lockdown), the summer of 2020 gave way to relaxations. However, scientific experts warned public authorities and the population of the danger of wide reopening during the summer, and indeed a strong second wave hit the country (BAG 2021b). Consequently, the Federal Council decided on a series of restrictions toward the end of October, which culminated with a second semi-lockdown decided by the Federal Council beginning January 18, 2021, including a home-office regulation and the closure of all nonessential businesses.

Switzerland is a highly decentralized federal country, with strong decision-making capacities of the cantons (subnational states). During the first wave, however, pandemic management was centralized at the national level. In fall 2020, the Federal Council refused to take the lead again and shifted the blame for the bad epidemic situation to the cantons, arguing that they had to enact their own restrictions depending on the local situation (Mavrot 2022). In this context, the way scientific evidence informed policymaking during the second wave was characterized by *medium salience, high credibility*, and *medium (to low) representativeness*.

#### Medium Salience

The Swiss National COVID-19 Science Task Force was rapidly established in March 2020 to ensure the provision of up-to-date medical expertise to the federal authorities. Members were nominated by the Federal Department of Home Affairs and the Federal Office of Public Health (FOPH) upon the proposition of the task force itself. Its members are top-level medical experts, active at universities and public hospitals. They serve on a voluntary basis. The structure and composition of the task force varied over time, but during the second wave, it included 10 expert thematic groups (e.g., immunology, clinical care, public health, data, and modeling), amounting to a total of 70 scientists (Mavrot 2022). The mission of the task force was not only to provide rough evidence to political and administrative authorities but also to formulate evidence-based recommendations.^17^ The task force was especially in contact with the experts of the national administration—in particular, the FOPH.

Strikingly, the expert system was strongly fitted for a centralized management of the pandemic at the national level, as Switzerland experienced during the first wave. However, with the partial shifting of pandemic management to the cantonal governments during the second wave, the system showed some limitations. The task force’s analyses of the epidemiological situation were mainly national, without differentiation of the regional situations.^18^ Moreover, although the task force was intended to be at the disposal of the cantons, few direct communication channels were established between the task force and the cantonal authorities. Consequently, the cantons also put in place various local expert advice systems.^19^ Hence, the salience of the expertise system was good regarding the prompt implementation of the task force and the regular delivery of up-to-date evidence and ready-made policy advice. However, it was medium regarding the fit of this organization to the overall politico-institutional federal system.

#### High Credibility

The credibility of the task force in the political and media scene was high. The task force was made up of the country’s top scientists, active both in research and in hospital practice, with a large majority being university professors. They were credited with a high degree of theoretical and frontline field knowledge throughout the crisis, which was not questioned in the public debates. The evaluation of the epidemiologic situation and the scientific reports of the task force complied with scientific standards and procedures (monitoring of daily new infections, calculation of R number, transmission rate of the various variants). Similarly, the policy briefs proposed recommendations based on projections or offered meta-analysis of the available scientific literature. The production of the task force relied on ad hoc studies and data analysis and not on general empirical assessments of the situation. The Federal Council and the federal health administration also gave consequent credit to the task force by regularly citing its work and recommendations during press conferences.

#### Medium to Low Representativeness

On the one hand, the representativeness of the task force’s scientists was high when considered from a strictly medical perspective. The large number of included experts allowed internal deliberations of the various dimensions of the pandemic within the scientific community. The collegial and internal deliberation of experts within the task force’s thematic groups allowed scientists to present a quite united front in their reports and policy papers. Because of the integrative nature of this system, there was little external criticism of the task force’s opinions from other scientists in the media. This collegial functioning was in line with the politico-institutional culture of the Swiss consensus-based democracy (Mavrot 2022). On the other hand, the task force was almost entirely limited to medical expertise, with a few exceptions (ethics, economics, law), which restricted its representativeness regarding other crucial aspects of the crisis. Furthermore, the fact that new experts were mainly selected by the task force itself through a co-optation mechanism led to discussions about the expert body’s representativeness.

The medium salience, the high credibility, and the medium to low representativeness as well as the composition of the task force puts it in the category of an evidence-based advice system. There were recurrent frictions between some scientists in the task force and national political authorities. While the Federal Council essentially followed the experts’ recommendations at the beginning of the first wave, the politics of the pandemic took another path at the end of the first lockdown with the reopening of economic activities (Sager and Mavrot 2020). During the second wave, national and cantonal authorities made several decisions that contradicted the opinion of the task force. Two moments of the pandemic management particularly triggered vivid disagreements between politicians and scientific experts. First, in addition to the criticized relaxations of the summer, the decision to reauthorize public events with more than 1000 persons (upon cantonal authorizations) beginning October 1, 2020, was one of them. The task force had warned in late August that such a decision would damage both public health and the economy (increase of quarantines).^20^ The political head of the Federal Department of Home Affairs later confessed to regretting this decision.^21^ Second, the late decision to enact a second semi-lockdown on January 18, 2021, was also a bone of contention and led to the resignation of one expert on the task force.^22^ The task force’s president made a clear policy recommendation based on the available evidence: “Our opinion is that we need very strong measures and geographically wide closings. The earlier the better.”^23^ Consequently, the experts on the task force publicly criticized the (lack of) decisions of the national authorities several times in the written press and on social media. The task force was then criticized for overstepping its competences and wanting to take on a political role despite its nonelective and advisory function.^24^ Right-wing governmental political parties tried to legally forbid the task force from directly communicating its opinions to the public, arguing that only the political authorities should do so.^25^ The proposal was rejected by the national parliament in March 2021. In conclusion, the task force provided a solid and up-to-date basis of evidence and advice throughout the pandemic, even achieving a good degree of salience at the national level for an evidence-based advice system. However, important aspects of its advice were ignored by the authorities during the second wave against the backdrop of a blame-shifting game between national and cantonal authorities.

### Italy

The second wave in Italy took place between early October 2020 and mid-January 2021, following a first wave with strong lockdown measures and a relaxation of restrictions during the summer (Chirico et al. 2021; ISTAT 2021). This second wave was characterized by a progressive reapplication of restrictions by national and regional governments, which from early November on was differentiated on a regional basis on the grounds of risk indicators (Malandrino and Capano 2022).

Following the first state of emergency declaration (council of ministers’ deliberation of January 31, 2020), the national executive was entrusted with substantial emergency management powers, although with leeway for regions to adopt more restrictive measures (Malandrino and Demichelis 2020). The bulk of decision-making at the central level was shared between the executive as a whole, the prime minister, the ministry of health, and the civil protection department (Malandrino et al. 2022). A few days after the state of emergency declaration, a Technical and Scientific Committee (CTS) was also established, which represented the main national-level policy advisory body during the pandemic (Capano 2020).^26^ In the second wave, the nature and role of policy advice was characterized by *medium-to-low representativeness, high salience*, and *medium credibility*.

#### Medium (to Low) Representativeness

The almost monopolistic presence of medical and healthcare professional backgrounds and knowledge in the CTS^27^ is a first confirmation of the limited representativeness of COVID-19–related policy advice. It also implied a subject-oriented analytical capacity (Howlett 2015). This in turn entailed the impossibility of examining “political and economic issues not within the competence of the CTS,”^28^ which was, however, partly moderated by the establishment of other issue-based expert task forces to support the work of ministries (Capano 2020; Galanti and Saracino 2021). In addition, a lack of usage of specific evidence by policymakers and the CTS was lamented by extra-CTS experts.^29^

#### High Salience

The CTS provided timely and relevant knowledge, often in the form of written dossiers containing the conclusions and policy recommendations stemming from the CTS meetings, sometimes even intervening on the very wording of to-be-adopted regulations. This indicates a direct involvement of experts in policy formulation.^30^ Discussions and consultations were frequent both between policymakers (e.g., between the prime minister and the ministry of health) and between these and the CTS, e.g., with regard to the restrictive measures to be adopted. The CTS frequently offered direct policy advice and suggestions rather than studies on which policymakers could base their decisions.

#### Medium Credibility

Among the members of the CTS are both practitioners and scientists, whose selection was based on both competence and prestige (Galanti and Lippi 2018). Despite this authoritativeness, the relationships between policy advice and politics were not always conflict-free. This was the case with the debate between Pierpaolo Sileri, vice-minister of health, and Luca Richeldi, an expert in respiratory diseases and a CTS member, during which the politician criticized the CTS for not being able to give a rapid and effective response to citizens on testing issues, while affirming that the CTS should be “at the service of politics and not vice versa.”^31^ Rather than indicating low scientific credibility, however, this conflict is emblematic of the distance between the logic of politics and policymaking, which require fast action to respond to pressing public demands, and the logic of science, which requires time to collect and elaborate data and to provide conclusions. While this led the CTS to play a role that is different from that of scientists, the available evidence points to the use by the CTS of externally conducted scientific studies and technical reports as well as constant monitoring of available data as grounds for their decisions, mediated by their own assessment of the situation.^32^

In sum, the Italian dynamics mostly confirm and partly adjust our expectations in terms of the operation of the three logics of scientific knowledge (Veit et al. 2017). The analyzed evidence points to a *medium to low representativeness* of the CTS, primarily jeopardized by the limited variety of backgrounds. In terms of credibility, while the CTS mostly provided direct policy advice and recommendations to policymakers, this content was often based on assessments of scientific data and studies and was communicated in written form. This pragmatic-but-science-based provision of policy advice was in turn recognized in terms of public credibility, which was additionally favored by the transparency concerning the CTS proceedings and members’ CVs. Strictly speaking, however, *scientific credibility was medium* due to the role of the CTS as a broker rather than a producer of knowledge. At the same time, this role represented a hint of the *high salience* of that advice, together with the rapidity with which the CTS was established after the first state of emergency declaration and the regularity of mutual consultation between the CTS and policymakers. Regarding the actual use of the CTS’s advice by policymakers, we can observe that the CTS often requested more stringent rules, the implementation of stricter enforcement by the authorities in charge of compliance control, or a clearer assumption of responsibilities by the competent authorities based on of-the-moment assessments of the epidemiological situation against the quality of the formal policies formulated and the effectiveness of their implementation.^33^ Still, the very fact that the CTS regularly contributed to the formulation of governmental policy—with policy recommendations issued on average every 3 days—legitimized not only governmental decisions at the national and regional levels^34^ but also private policy actors’ positions. For instance, the lawyer of a football team advocated for a change in the rules by stating that this was recommended “by the entire technical–scientific community.”^35^ Nevertheless, policymakers did not always follow the CTS’s recommendations, as was the case with transportation policy. The CTS had long been advocating for measures to avoid crowds on public transport, especially at peak times.^36^ However, there was a gap between such policy advocacy/advice (and the perception of its significance by its proponents) and the policies actually adopted.^37^ Overall, compared with the preexisting framework in which scientific knowledge found relatively little place in a system dominated by a legalistic culture, the establishment and regular consultation of the CTS represented a new phenomenon. This phenomenon configures the typical definition of policy advice as boundary work between science and policy (Galanti and Lippi 2018) and entails a clear distinction between the CTS’s activities and those typical of ministerial cabinets as internal policy advisors (Di Mascio and Natalini 2013). Only time will tell if the Italian policymaking system will be able to learn from this experience and enter a new era of science–policy relationships.

## Comparative Discussion

The theory section holds two sets of assumptions: first regarding the manifestation of policy advisory systems during a crisis in the three test countries, and second regarding their appropriateness for delivering relevant advice during a crisis.

Comparing the three systems of policy advice in Germany, Switzerland, and Italy, we first find only partial confirmation of our expected features of expertise-focused and evidence-focused systems. Table 2 summarizes the three cases. Germany, as a case of expert-based advice, shows high salience of offered knowledge, low to medium credibility, and low representativeness. All three logics largely conform to the open expectation. Switzerland, as a case of evidence-focused advice, displays medium salience (as opposed to low salience outside the COVID-19 crisis), high credibility as expected, and medium to low representativeness. The case thus differs in two respects from our typology derived from theory, both of which are due to situational and institutional idiosyncrasies. Italy, as a case of expert advice, largely conforms to and partly adjusts the expectations: Salience is high, as expected; credibility is medium, whereas it was expected to be low because of the timing of emergency policymaking; and representativeness is medium to low, whereas the expectation was open.

**Table 2:** Contrasting cases and expectations

Logic of scientific knowledge	Salience	Credibility	Representativeness	Adoption of expert advice into policy
Germany: Expertise-focused policy advice system	High (exp. high)	Low to medium (exp. low)	Low (exp. low–high)	Largely yes
Switzerland: Evidence-focused policy advice system	Medium (exp. low)	High (exp. high)	Medium (to low) (exp. high)	Moderate (in important parts not)
Italy: Expertise-based policy advice system	High (exp. high)	Medium (exp. low)	Medium (to low) (exp. low–high)	Largely yes
Assumed demands on scientific knowledge in times of crisis	High	Low–moderate	Low–moderate	Adoption

The cases imply that existing expert-based knowledge logics prevail during crises, whereas this is not the case for the Swiss system, which relies on evidence rather than expertise. This finding implies that the two expert-based systems were better prepared for the sudden increase in the need for new knowledge in a situation of uncertainty. The system based on evidence, in contrast, needed adaptation, which meant disruption from the established structure. This disruption suggests that the produced knowledge was less well received in the adapted evidence-focused system of Switzerland than in the other two countries. We discuss this second question in the next paragraph.

As Table 2 shows, the offered knowledge during the second wave made its way into policy in all three countries. However, the governments in Germany and Italy adopted scientific advice to a larger extent than the Swiss government did. It is difficult to argue a clear causal link between the produced knowledge and its adoption because we have established deeply different institutional designs of scientific advice that are prone to determine both the produced knowledge and its use. In addition, the crisis had its idiosyncrasies in all three countries under scrutiny. Still, the finding that the Swiss advisory system provides advice that seems to have less utility for politics than the advice provided in Germany and Italy with expert-based systems corroborates our initial argument. The transfer from science to politics runs more smoothly in the latter two countries because of the established routine at the intersection between experts and politicians in institutionalized structures. In Switzerland, these structures are amiss. The system relies on tailored evidence production but not on a routine exchange between experts and politics. With the COVID-19 crisis, Switzerland experienced a shift in the policy advice system. While advice had been based on policy evaluations or extraparliamentary commissions before the pandemic, the crisis gave an unprecedented weight to medical scientists gathered in an ad hoc college, without the preexistence of a collaboration culture between them and the politicians. Moreover, the COVID-19 policy advice gave way to high personalization of the scientific advice through the task force’s leaders, which is an uncommon feature of the consensus- and coalition-based Swiss governance style and added to the tensions between science and politics. The recipient side had little experience in dealing with expert advice, and experts had little experience in providing policy advice. As a result, scientific advice had less weight in the Swiss system, where it had to compete with important vested interests and party politics. In Germany and Italy, the established structures better allowed for the provision of relevant expertise and its usage. Especially in Germany, the preexisting channels of expert-based policy advice were activated.

## Conclusion

This study raises the question of how established structures of scientific advice foster EBP in times of crisis. We employ the distinction of expertise vs. evidence as a defining structure of advisory systems and three features of knowledge for the mapping of the form and content of policy advice in Germany, Switzerland, and Italy. We find that Germany’s and Italy’s expert-based advisory systems are better suited to integrate relevant scientific advice into policymaking in times of crisis than the evidence-focused system that we found in Switzerland. These differences in our observed outcome (i.e., the use of scientific knowledge in policymaking), however, are not necessarily crucial for the way the three countries came through the second wave at large (e.g., Switzerland had a significantly higher infection rate but did not fare worse in terms of fatalities than the two other countries). However, those differences in integrating scientific advice in policy decisions during the pandemic do tell us something if we observe the knowledge dimensions’ departures from our expectations, namely in terms of credibility. They indicate that credibility can be a matter of perception in relation to the preexisting policy advice system: In systems in which scientific evidence (with high credibility) had rarely been embedded in policymaking before the pandemic, its comparatively higher usage in times of crisis might generate a perception of high credibility. Consequently, policymakers might use that knowledge to legitimize their own actions, as occurred in the cases of Italy and Germany. In Switzerland, policymakers also strongly used scientific advice during the COVID-19 crisis to legitimize their decisions when needed, but such strategic use of evidence is also usual in noncrisis times with policy evaluations.

These findings bear important practical insights. It is well known that EBP does not come out of the blue. The three cases show how institutional structures can foster both the provision and the use of scientific advice. Established cooperation between experts and politicians makes it easier to refer to these routines in times of acute need for knowledge in times of sudden uncertainty. On the downside, this means that EBP that only refers to the production of tailored evidence for specific policy questions does not suffice as a structure firm enough for knowledge transfer in times of acute crisis. The expert–politician nexus is a social interface that needs established forms of exchange and a good fit with the politico-institutional features of the system. In order to establish these structures, institutions are the adequate way to build and maintain a routine that decision-makers can refer to in times of acute crisis.

## Supplementary Information

The online annex provides detailed information on the sources analyzed in the case studies. Table A1 summarizes the newspapers consulted in each country. Additionally, the annex includes a list of all press and media sources consulted. Table A2 outlines the indicator questions asked to operationalize the three logics of scientific knowledge (salience, credibility, representativeness). Table A3 summarizes the alignment of expert recommendations with decisions taken at governmental meetings in Germany.

## Footnotes

1 Italian Law no. 50/1999.

2 G1.

3 G1.

4 G2.

5 G3, G4.

6 G4.

7 In 2020, the national academy of science alone published seven ad hoc statements on COVID-19 with explicit guidance for policymakers: https://www.leopoldina.org/uploads/tx_leopublication/2020_Leopoldina-Stellungnahmen_Coronavirus-Pandemie_1-7.pdf [accessed September 8, 2021].

8 G3.

9 G5.

10 https://www.gruene-bundestag.de/themen/gesundheit/pandemierat-jetzt-gruenden; https://dserver.bundestag.de/btd/19/252/1925254.pdf [accessed September 8, 2021].

11 G6.

12 G7.

13 G8.

14 Since these recurring meetings of federal and subnational decision-makers are a peculiarity of the German context, no comparable data can be gathered for the other two countries studied. For full transparency, we still consider it beneficial for the reader to list the three meetings and the related expert recommendations in the online annex.

15 G9.

16 G1.

17 https://sciencetaskforce.ch/en/mandate/.

18 Up to some rare exceptions; see, e.g., https://sciencetaskforce.ch/fr/policy-brief/analyse-des-differences-regionales-durant-la-deuxieme-vague/.

19 E.g., S1.

20 https://sciencetaskforce.ch/fr/policy-brief/quels-compromis-entre-sante-et-economie/; also S2.

21 S3.

22 E.g., S4 and S5.

23 S6 (translation).

24 S7.

25 S8.

26 Decree of the Head of the Civil Protection Department of the Presidency of the Council of Ministers (HCPDCMP) no. 371, February 5, 2020.

27 HCPDCMP ordinances, April 18, May 15, and November 25, 2020.

28 CTS proceedings, August 26, 2020, translation.

29 I3.

30 E.g., CTS proceedings, October 24, 2020.

31 I11.

32 E.g., CTS proceedings, November 27, 2020, and I8.

33 E.g., I9.

34 E.g., I1, I2, I5 or I10.

35 I4.

36 E.g., CTS proceedings of April 18, August 26, and September 7, 2020.

37 E.g., I6.
