# Supplementary material for: Evidence-Based Policymaking in Times of Acute Crisis: Comparing the Use of Scientific Knowledge in Germany, Switzerland, and Italy
Source: Polit Vierteljahresschr. 2022 Apr 4;63(2):359–82. doi: 10.1007/s11615-022-00382-x (PMC8977835; doi:10.1007/s11615-022-00382-x)
Supplement: Supplementary file 1 — The online annex provides detailed information on the sources analyzed in the case studies. Table A1 summarizes the newspapers consulted in each country. Additionally, the annex includes a list of all press and media sources consulted. Table A2 outlines the indicator questions asked to operationalize the three logics of scientific knowledge (salience, credibility, representativeness). Table A3 summarizes the alignment of expert recommendations with decisions taken at governmental meetings in Germany. [file 11615_2022_382_MOESM1_ESM.docx]

**Online Annex**

***Evidence-based Policymaking in Times of Acute Crisis: Comparing the Use of Scientific Knowledge in Germany, Switzerland and Italy***

***Susanne Hadorn,*** *KPM Center for Public Management, University of Bern, Switzerland*

*Email:* [*susanne.hadorn@kpm.unibe.ch*](mailto:susanne.hadorn@kpm.unibe.ch)

*ORCID: 0000-0002-4470-7708*

***Fritz Sager,*** *KPM Center for Public Management, University of Bern, Switzerland*

*Email:* [*fritz.sager@kpm.unibe.ch*](mailto:fritz.sager@kpm.unibe.ch)

*ORCID: 0000-0001-5099-6676*

***Céline Mavrot,*** *Institute of Social Sciences, University of Lausanne, Switzerland*

*Email:* [*celine.mavrot@unil.ch*](mailto:celine.mavrot@unil.ch)

*ORCID: 0000-0001-9603-5790*

***Anna Malandrino,*** *KPM Center for Public Management, University of Bern, Switzerland and Department of Political and Social Sciences, University of Bologna, Italy*

*Email:* [*anna.malandrino@kpm.unibe.ch*](mailto:anna.malandrino@kpm.unibe.ch)

*ORCID: 0000-0002-3643-9600*

***Jörn Ege,*** *KPM Center for Public Management, University of Bern, Switzerland and University of Konstanz, Germany*

*Email:* [*joern.ege@kpm.unibe.ch*](mailto:joern.ege@kpm.unibe.ch)

*ORCID:* *0000-0001-7286-6880*

**Table A1:** *Details of the data sources for the three cases*

| **Country** | **Major sources** |
| --- | --- |
| Germany | In order to triangulate and cross-validate the information from the different data sources, we included two major German newspapers (from both sides of the political spectrum), other (online) media outlets and official documents published by the German government. More specifically, we focus on articles in two major newspapers: the left-liberal “Süddeutsche Zeitung” and the conservative-liberal “Frankfurter Allgemeine Zeitung”. We also used LexisUni to supplement our findings with reports in other newspapers (see list of sources below) (especially the left-alternative “tageszeitung” and more populist online newspaper “Bild.de”). We focused our research on the eight experts that were present during the meeting with the Chancellor and the heads of the Länder governments on January 4, 2021 (see G4). |
| Switzerland | To reconstitute both the expert advice and the political activity, the Swiss case triangulates the governmental decisions, the analyses and recommendations produced by the scientific advisors, and media accounts of the events. The data includes the governmental decisions at the federal level through the successive modifications of the COVID-19 legislation. The data also comprises the documentation produced by the specialized ad-hoc expert group put in place by the Swiss government at the national level (the Federal Council) in face of the pandemic: *the Swiss National COVID-19 Science Task Force*. This documentation consists of periodic reports (“Evaluation of the epidemiologic situation” and “Scientific reports”) and regular position papers on specific topics such as mask policy or measures at the borders (“Policy briefs”). In addition, two leading newspapers were analyzed: the *Neue Zürcher Zeitung* (German-speaking, liberal) and *Le Temps* (French-speaking, progressive), with a total of 80 screened articles. |
| Italy | Source triangulation for the Italian case concerned media sources (two major Italian newspapers with different orientations + other media sources identified through a snowballing process), official policy advice documents and the scientific studies mentioned therein, as well as adopted measures. In particular, the documents analyzed for the Italian case consisted of meeting proceedings and recommendations issued by policy advisors (in particular by the COVID-19 Technical and Scientific Committee), scientific studies employed in policymaking processes and governmental decisions. In addition, public declarations were extracted from the two major national newspapers, i.e. *Corriere della Sera* (with a liberal orientation) and *La Repubblica* (with a progressive orientation), for a total of respectively 251 and 190 screened articles. Starting from such press coverage, videos with public declarations by policymakers and advisors, published by several broadcasting channels, were also considered. |

**Table A2:** *Indicator question to operationalize the three logics of scientific knowledge*

| **Logics of knowledge** | **Indicator questions** |
| --- | --- |
| Salience | Did the consulted experts formulate direct policy advice, or did they only provide rough evidence overviews?  How quickly was the COVID-19 expert advice system put in place?  How close were the experts to the politicians (frequency of meetings if any, communication flows and means)? |
| Credibility | Did the experts rely on their personal assessment of the situation or on ad hoc studies of the situation?  Did the production of expertise comply with scientific standards and procedures?  Were the consulted experts scientists or practitioners? |
| Representativeness | How diverse was the profile of the consulted experts?  Was there public criticism against the composition of the expert groups?  Did any expert excluded from the consultation system have to express their criticism in the media? |

**Table A3:** *Alignment of expert recommendations with core decisions at government meetings in Germany*

| **Core decisions** | **Expert recommendations** | **Expert followed?** |
| --- | --- | --- |
| Conference 28 October 2020  *soft lockdown*  *shops, daycare and schools remain open*. | Wieler: restrictions of private gatherings to max. 10 people from two households; ban of private parties in public spaces; achieve more coherence of rules across Länder; no hard lockdown necessary. Schools are unlikely to be drivers of the pandemic and can remain open.  Leopolina (ad hoc statement from Sept. 23): binding, federally coherent and effective measures but the goal should be to keep public and economic life as unrestricted as possible.  Drosten (together with Lauterbach): early lockdown, with school and stores to remain open; increased tracing of outbreak clusters.  Meyer-Hermann: preventive measures such as mandatory masks and fines but also considers mandatory travel restrictions for people in high-risk areas.  Brinkmann (together with Drosten and others): strongly recommended not to waive Corona restrictions to achieve herd immunity (as requested in the Great Barrington Declaration) | Wieler: followed  Leopoldina: followed  Drosten: mostly followed (but no mention of increased cluster tracing)  Meyer-Hermann: mostly followed (but travel restrictions only encouraged)  Brinkmann: followed |
| Conference 13 December 2020  *hard lockdown with temporary easing of measures over Christmas* | Wieler: continuation of measures (and more restrictions in nursing homes); recommends keeping benchmark of 7-day incidence rate at 50. If numbers do not fall, no other option than a hard lockdown with no relaxations over Christmas.  Leopoldina (ad hoc statement 8 December): recommends hard lockdown.  Drosten: recommends keeping schools closed or highly restricted.  Brinkmann: called for measures to break 2^nd^ wave already in early November. | Wieler: mostly followed (but government still eased measures over Christmas)  Leopoldina: followed  Drosten: followed  Brinkmann: followed (but with delay) |
| Conference 5 January 2021  *extension of hard lockdown, but no tightening of measures* | Wieler: recommends continuation of social distancing measures and other restrictions  Experts at consultation meeting with governments: recommend continuation or even tightening of lockdown measures.  Berner at consultation meeting with governments: recommends opening of schools (under strict hygiene measures) because his study shows that most infections happen outside of schools. | Wielder: followed  Consulted experts: mostly followed  Berner: evidence not followed |

**List of press and media sources consulted**

**1. Germany:**

Sources directly relevant to the main article:

*G1:* Oltermann, Phillip. 2020. Compromise lockdown struggles to subdue Germany's Covid second wave. *The Guardian.* 10 December 2020. https://www.theguardian.com/world/2020/dec/10/compromise-lockdown-struggles-to-subdue-germany-covid-second-wave. Accessed 28 September 2021.

G2: tagesschau. 2020. Corona-Pandemie: Auf wen die Bundesregierung hört. *tagesschau.de.* 20 October 2020. https://www.tagesschau.de/inland/coronavirus-bundesregierung-berater-101.html. Accessed 28 September 2021.

G3: Römer, Jörg and Julia Merlot. 2021. Corona-Shutdown: Diese acht Fachleute beraten Bundesregierung und Länderchefs. *Der Spiegel.* 18 Januar 2021. https://www.spiegel.de/wissenschaft/medizin/coronavirus-diese-sieben-fachleute-beraten-bundesregierung-und-laenderchefs-a-93abc4f5-cac1-4cbb-bc22-8d3b9c623b28. Accessed 29 September 2021.

G4: Aswad, Nadja and Julius Böhm. 2021. Lockdown – DAS raten die Top-Wissenschaftler wie Prof. Drosten der Kanzlerin. *Bild.* 5 Januar 2021. https://www.bild.de/bild-plus/politik/inland/politik-inland/lockdown-das-raten-die-top-wissenschaftler-wie-prof-drosten-der-kanzlerin-74743730,view=conversionToLogin.bild.html. Accessed 28 September 2021.

*G5: taz, die tageszeitung*. 2020. Fehlgerechnet; Die Politik hat sich bei den Anti-Corona-Maßnahmen auf den Rat von zu wenigen Fachleuten gestützt. Nötig ist ein breit aufgestelltes Expertengremium. *Taz, die Tageszeitung.* 10 August 2020. https://advance.lexis.com/api/document?collection=news&id=urn:contentItem:60JF-9PY1-JDJ5-S2JR-00000-00&context=1516831. Accessed 28 September 2021.

G6: Hufnagel, Margit. 2021. RKI-Chef Wieler erlebt massive Anfeindungen. *Augsburger Allgemeine.* 11 August 2021. https://www.augsburger-allgemeine.de/politik/Corona-Pandemie-RKI-Chef-Wieler-erlebt-massive-Anfeindungen-id60312321.html. Accessed 8 September 2021.

G7: Katja Thorwarth. 2021. Hendrik Streeck über Corona-Risiko bei Fußball-EM und Olympia: „Das haben wir nicht untersucht“. *Frankfurter Rundschau.* https://www.fr.de/wissen/virologe-hendrik-streeck-corona-pandemie-deutschland-fussball-europa-meisterschaft-90189921.html. Accessed 8 September 2021.

G8: Andreas Speit. 2021. Impfskepsis und Antisemitismus. *Taz, die Tageszeitung.* 21 July 2021. https://taz.de/Die-Basis-Kandidat-Sucharit-Bhakdi/!5781717/. Accessed 8 September 2021.

G9: tagesschau. 2020. Generaldebatte im Bundestag: Merkel dringt auf harten Lockdown. *tagesschau.de.* 9 December 2020. https://www.tagesschau.de/inland/merkel-corona-generaldebatte-101.html. Accessed 24 September 2021.

Sources not explicitly referred to in the main article:

Süddeutsche Zeitung. 2020. Den Infizierten auf der Spur. *Süddeutsche Zeitung.* 26 October 2020. https://projekte.sueddeutsche.de/artikel/wissen/den-infizierten-auf-der-spur-e282396/?reduced=true. Accessed 27 September 2021.

Süddeutsche Zeitung. 2020. Corona News: Spahn will Vertrag mit Biontech/Pfizer. 9 November 2020. https://www.sueddeutsche.de/politik/coronavirus-news-impfstoff-biontech-pfizer-spahn-1.5101677?reduced=true. Accessed 28 September 2021.

Süddeutsche Zeitung. 2020. RKI meldet 10 824 Neuinfektionen. 16 November 2020. https://www.sueddeutsche.de/politik/coronavirus-news-rki-beschraenkungen-1.5109218?reduced=true. Accessed 28 September 2021.

Süddeutsche Zeitung. 2020. Corona: RKI meldet knapp 11 000 Neuinfektionen. 23 November 2020. https://www.sueddeutsche.de/politik/corona-news-1.5116448?reduced=true. Accessed 28 September 2021.

Süddeutsche Zeitung. 2020. Corona News: RKI meldet 12 332 Neuinfektionen. 7 December 2020. https://www.sueddeutsche.de/politik/corona-news-rki-neuinfektionen-1.5132169?reduced=true. Accessed 28 September 2021.

Süddeutsche Zeitung. 2020. RKI registriert 16 362 Neuinfektionen. 14 December 2020. https://www.sueddeutsche.de/politik/corona-news-rki-1.5139667?reduced=true. Accessed 28 September 2021.

Süddeutsche Zeitung. 2020. Forscherinnen fordern europäische Strategie gegen die Corona-Pandemie. 19 December 2020. https://www.sueddeutsche.de/politik/corona-gesundheitspolitik-priesemann-1.5153123?reduced=true. Accessed 28 September 2021.

Süddeutsche Zeitung. 2020. Corona-Zahlen: Wie blicken Sie auf die Feiertage? 22 December 2020. https://www.sueddeutsche.de/leben/coronavirus-weihnachten-feiertage-rki-1.5155970. Accessed 28 September 2021.

Süddeutsche Zeitung. 2020. RKI: Wieler warnt vor Weihnachtstreffen. 22 December 2020. https://www.sueddeutsche.de/politik/corona-news-rki-neuinfektionen-1.5154243?reduced=true. Accessed 28 September 2021.

Süddeutsche Zeitung. 2020. Mehr als 30 000 Corona-Tote in Deutschland. 28 December 2020. https://www.sueddeutsche.de/gesundheit/corona-news-deutschland-rki-neuinfektionen-spahn-1.5155887?reduced=true. Accessed 28 September 2021.

Bergmann, Julia and Constanze von Bullion. 2020. „Die Situation ist sehr ernst“. *Süddeutsche Zeitung.* 22 October 2020. https://www.sueddeutsche.de/politik/corona-krise-die-situation-ist-sehr-ernst-1.5090672. Accessed 27 September 2021.

Bundestagsfraktion, Bündnis 90/Die Grünen. 2021. Pandemierat jetzt gründen. https://www.gruene-bundestag.de/themen/gesundheit/pandemierat-jetzt-gruenden. Accessed 8 September 2021.

Deutscher Bundestag. 2020. *Drucksache 19/25254 : Gesetzentwurf der Abgeordneten Amira Mohammed Ali, Dr. Dietmar Bartsch, Jan Korte, Petra Pau, Friedrich - Straetmanns, Dr. André Hahn, Gökay Akbulut, Ulla Jelpke, Niema Movassat, Martina Renner, Kersten Steinke und der Fraktion DIE LINKE. Entwurf eines Gesetzes für einen Pandemierat des Bundestages (Pandemieratgesetz - PandemieratG).*

Deutscher Bundestag. 2020. *Drucksache 19/25658 : Antwort der Bundesregierung auf die Kleine Anfrage der Abgeordneten Detlev Spangenberg, Dr. Robby Schlund, Dr. Axel Gehrke, weiterer Abgeordneter und der Fraktion der AfD– Drucksache 19/25144 – Beratung der Bundesregierung durch medizinische Fachgesellschaften in Fragen des Infektionsschutzes gegen das Coronavirus*.

Deutscher Bundestag. 2021. *Drucksache 19/26787 : Antwort der Bundesregierung auf die Kleine Anfrage der Abgeordneten Fabio De Masi, Jörg Cezanne, Klaus Ernst, weiterer Abgeordneter und der Fraktion DIE LINKE.– Drucksache 19/25452 – Ausgaben für Beratungs- und Unterstützungsleistungen während der Corona-Krise*.

Endt, Christian. 2020. Exponentielles Wachstum, exponentielles Schrumpfen. *Süddeutsche Zeitung.* 18 December 2020. https://www.sueddeutsche.de/wissen/coronavirus-infektionszahlen-lockdown-1.5152676?reduced=true. Accessed 28 September 2021.

Endt, Christian. 2020. Coronavirus: Die Gefahr reist im Weihnachtsurlaub mit. *Süddeutsche Zeitung.* 30 December 2020. https://www.sueddeutsche.de/politik/coronavirus-reise-regeln-1.5162205?reduced=true. Accessed 28 September 2021.

Felix Lee. 2021. Viele offene Fragen; Kinder haben bei Corona-Infektionen zwar oft keine Symptome. Das Virus können sie aber trotzdem an Erwachsene weitertragen. Ob Schulen Treiber der Pandemie sind, wird weiterhin heiß diskutiert. *taz, die tageszeitung.* 6 Januar 2021. https://advance.lexis.com/api/document?collection=news&id=urn:contentItem:61P7-6421-JDJ5-S0MY-00000-00&context=1516831. Accessed 28 September 2021.

Frankfurter Allgemeine Zeitung. 2020. Einschränkungen sollen bleiben – auch mit Impfstoff. *Frankfurter Allgemeine Zeitung.* 13 October 2020. https://www.faz.net/aktuell/politik/inland/rki-trotz-corona-impfstoff-werden-einschraenkungen-bleiben-16999260.html. Accessed 27 September 2021.

Frankfurter Allgemeine Zeitung. 2020. „Es geht hier gerade um die Wurst“. *Frankfurter Allgemeine Zeitung.* 16 October 2020. https://www.faz.net/aktuell/gesellschaft/gesundheit/coronavirus/corona-michael-meyer-hermann-warnt-vor-ausbreitung-17004354.html. Accessed 27 September 2021.

Frankfurter Allgemeine Zeitung. 2020. RKI warnt vor starkem Anstieg in Alten- und Pflegeheimen. *Frankfurter Allgemeine Zeitung.* 3 December 2020. https://www.faz.net/aktuell/politik/inland/rki-zu-viele-infektionen-mit-coronavirus-in-deutschland-17082843.html. Accessed 27 September 2021.

Frankfurter Allgemeine Zeitung. 2020. RKI: Corona-Lage verschlechtert sich. *Frankfurter Allgemeine Zeitung.* 10 December 2020. https://www.faz.net/aktuell/gesellschaft/gesundheit/coronavirus/rki-corona-lage-in-deutschland-verschlechtert-sich-17095045.html. Accessed 28 September 2021.

Frankfurter Allgemeine Zeitung. 2020. Bund will Impfstofflieferungen vorziehen. *Frankfurter Allgemeine Zeitung.* 30 December 2020. https://www.faz.net/aktuell/politik/inland/bund-kuendigt-an-impfstofflieferungen-vorzuziehen-17124789.html. Accessed 28 September 2021.

Frankfurter Allgemeine Zeitung. 2021. Lorz legt Papier für Schulen vor. *Frankfurter Allgemeine Zeitung.* 5 Januar 2021. https://www.faz.net/aktuell/rhein-main/lockdown-verlaengerung-wie-es-mit-der-schule-weitergeht-17130827.html. Accessed 28 September 2021.

Fried, Nico. 2020. Schlupfloch-Parade. *Süddeutsche Zeitung.* 14 October 2020. https://www.sueddeutsche.de/politik/droht-ein-neuer-lockdown-schlupfloch-parade-1.5068360. Accessed 27 September 2021.

Fried, Nico. 2020. Corona-Maßnahmen: Lange Verhandlungen im Kanzleramt. *Süddeutsche Zeitung.* 15 October 2020. https://www.sueddeutsche.de/politik/corona-deutschland-massnahmen-merkel-1.5070761?reduced=true. Accessed 27 September 2021.

Köhn, Rüdiger and Timo Kotowski. 2020. Wie es für den deutschen Tourismus nun weitergeht. *Frankfurter Allgemeine Zeitung.* 24 October 2020. https://www.faz.net/aktuell/wirtschaft/mehr-wirtschaft/wie-es-fuer-den-deutschen-tourismus-nun-weitergeht-17016877.html. Accessed 27 September 2021.

Köppe, Julia. 2020. Zu hohe Infektionszahlen: Wissenschaftler fordern neue Corona-Strategie für Europa. *Der Spiegel.* 18 December 2020. https://www.spiegel.de/wissenschaft/medizin/corona-hochrangige-wissenschaftler-fordern-richtwert-von-maximal-zehn-neuinfektionen-fuer-europa-a-588598e8-7533-4193-b4cd-c9c01f7aeaf8. Accessed 8 September 2021.

Leopoldina - Nationale Akademie der Wissenschaften. 2020. Ad-hoc-Stellungnahmen zur Coronavirus-Pandemie. https://www.leopoldina.org/uploads/tx_leopublication/2020_Leopoldina-Stellungnahmen_Coronavirus-Pandemie_1-7.pdf. Accessed 8 September 2021.

Lohse, Eckart and Heike Schmoll. 2020. Darauf haben sich Bund und Länder geeinigt. *Frankfurter Allgemeine Zeitung.* 14 October 2020. https://www.faz.net/aktuell/politik/inland/corona-gipfel-darauf-haben-sich-bund-und-laender-geeinigt-17002193.html. Accessed 27 September 2021.

Ludwig, Kristiana. 2020. Merkel will am Mittwoch über neue Regeln beraten. *Süddeutsche Zeitung.* 26 October 2020. https://www.sueddeutsche.de/politik/corona-pandemie-merkel-will-am-mittwoch-ueber-neue-regeln-beraten-1.5094710. Accessed 27 September 2021.

Piatov, Filipp. 2020. Drosten Vorschlag für Ü-50-Kennzahl bekommt Absage der Regierung. *Bild.* 21 October 2020. https://www.bild.de/bild-plus/politik/inland/politik-inland/drosten-vorschlag-fuer-ue-50-kennzahl-bekommt-absage-der-regierung-73526118,view=conversionToLogin.bild.html. Accessed 28 September 2021.

Roßbach, Henrike. 2020. Gefahr für Senioren. *Süddeutsche Zeitung.* 3 December 2020. https://www.sueddeutsche.de/politik/pflegeheime-in-der-pandemie-gefahr-fuer-senioren-1.5136471?reduced=true. Accessed 28 September 2021.

Schmoll, Heike. 2020. Die meisten Schüler stecken sich nicht im Klassenzimmer an. *Frankfurter Allgemeine Zeitung.* 19 November 2020. https://www.faz.net/aktuell/politik/inland/corona-in-schulen-infektion-meist-nicht-im-klassenzimmer-17060553.html. Accessed 28 September 2021.

Zinkant, Kathrin. 2020. Durchseuchung wäre eine Katastrophe. *Süddeutsche Zeitung.* 20 October 2020. https://www.sueddeutsche.de/gesundheit/coronavirus-deklaration-massnahmen-drosten-1.5085207?reduced=true. Accessed 27 September 2021.

Zinkant, Kathrin. 2020. Corona in der Schule. *Süddeutsche Zeitung.* 4 November 2020. https://www.sueddeutsche.de/gesundheit/corona-schule-kinder-lockdown-ansteckungen-1.5103385?reduced=true. Accessed 28 September 2021.

**2. Switzerland:**

S1: Perrin, Léa. 2020. Vaud se dote d'un conseil scientifique Covid-19. *Le Temps.* 8 October 2020. <https://www.letemps.ch/suisse/vaud-se-dote-dun-conseil-scientifique-covid19>. Accessed 28 September 2021.

S2: Gerny, Daniel. 2020. Epidemiologe Marcel Tanner: „An Grossveranstaltungen ist derzeit nicht zu denken“. *Neue Zürcher Zeitung*. 30 July 2020. Accessed 28 September 2021.

S3: Le Temps. 2021. Mea culpa d’Alain Berset qui interroge le rapport du Conseil fédéral aux experts scientifiques. 21 May 2021. <https://www.letemps.ch/suisse/mea-culpa-dalain-berset-interroge-rapport-conseil-federal-aux-experts-scientifiques>. Accessed 28 September 2021

S4: Stalder, Helmut. 2020. Die Experten drängen auf einen landesweiten Teil-Lockdown – streiten sich aber über den Zeitpunkt. *Neue Zürcher Zeitung.* 15 November 2020. <https://www.nzz.ch/schweiz/covid-task-force-empfiehlt-einen-landesweiten-teil-lockdown-ld.1587115?reduced=true>. Accessed 28 September 2021.

S5: *Petignat, Yves. 2021.* Le savoir contre le pouvoir. *Le* *Temps.* 17 January 2021. <https://www.letemps.ch/opinions/savoir-contre-pouvoir>. Accessed 28 September 2021.

S6: Rhyn, Larissa, Fabian Schäfer, and Daniel Gerny. Fachleute sind in Alarmstimmung – doch Bundesrat Berset wartet ab. *Neue Zürcher Zeitung.* 15 December 2020. <https://www.nzz.ch/schweiz/coronavirus-fachleute-in-alarmstimmung-doch-berset-wartet-ab-ld.1592260?reduced=true>. Accessed 28 September 2021.

S7: Nock, Yannick. 2021., «Überschätzte Wissenschaft? „Experten machen Politik, statt zu beraten – so geht das nicht“. *Neue Zürcher Zeitung*. 22 March 2021. <https://www.nzz.ch/international/ueberschaetzte-wissenschaft-experten-machen-politik-statt-zu-beraten-so-geht-das-nicht-ld.1607115?reduced=true>. Accessed 28 September 2021.

S8: Le Temps avec les agences. 2021. Au terme de la plus longue séance de son histoire, le Conseil national refuse de presser l'ouverture des restaurants au 22 mars: les nouvelles du 8 mars. *Le Temps*. 8 March 2021. <https://www.letemps.ch/monde/terme-plus-longue-seance-histoire-conseil-national-refuse-presser-louverture-restaurants-22>. Accessed 28 September 2021.

**3. Italy:**

Screened sources totals (due to the high number of sources consulted it is impossible to provide all the references here):

- from *La Repubblica* newspaper: 251 articles
- from *Corriere della Sera* newspaper: 190 articles
- other media (collected through snowballing technique): videos from TVRep, Rai 3, La7

Sources directly relevant to the main article:

I1: Marchese, Domenico. 2020. I tamponi, l'intervento della Asl, lo scontro tra De Laurentiis e la Lega: così è scoppiato il caos di Juve-Napoli. *La Repubblica*. 5 October 2020. <https://www.repubblica.it/sport/calcio/serie-a/2020/10/05/news/juventus_napoli_cronistoria_caos_asl_lega-269532557/?ref=search>. Accessed 13 October 2021.

I2: Zunino, Corrado. 2020. Azzolina: a scuola pochi contagi "Ma nelle Regioni babele di regole. *La Repubblica.* 6 October 2020.<https://ricerca.repubblica.it/repubblica/archivio/repubblica/2020/10/06/azzolina-a-scuola-pochi-contagi-ma-nelle-regioni-babele-di-regole04.html?ref=search>. Accessed 13 October 2021.

I3: Giannoli, Viola. 2020. Il microbiologo Crisanti: “Il mio piano sui tamponi ignorato. Mesi buttati e ora piangiamo”. *La Repubblica.* 7 October 2020. [https://ricerca.repubblica.it/repubblica/archivio/repubblica/2020/10/08/crisantiil-mio-piano-ignorato-mesi-buttati-e-ora-piangiamo04.html?ref=search](https://ricerca.repubblica.it/repubblica/archivio/repubblica/2020/10/08/crisantiil-mio-piano-ignorato-mesi-buttati-e-ora-piangiamo04.html?ref=search%C3%AC). Accessed 13 October 2021.

I4: La Repubblica. 2020. Il legale Mattia Grassani, avvocato del Napoli: domani la sentenza sul caso Juve. *La Repubblica.* 13 October 2020.<https://ricerca.repubblica.it/repubblica/archivio/repubblica/2020/10/13/grassaniil-napoli-non-e-partito-per-non-violare-la-leggeNapoli14.html?ref=search>. Accessed 13 October 2021.

I5: La Repubblica. 2020. Basket, calcio e skate il Comune chiude 72 aree per lo sport. *La Repubblica*. 17 October 2020.<https://ricerca.repubblica.it/repubblica/archivio/repubblica/2020/10/17/basket-calcio-e-skate-il-comune-chiude-72-aree-per-lo-sportFirenze02.html?ref=search>. Accessed 13 October 2021.

I6: Sarzanini, Fiorenza. 2020. Miozzo (Cts): “Rispettare le regole anti-Covid per due settimane o si chiude tutto”. *Corriere della Sera*. 29 October 2020.<https://www.corriere.it/cronache/20_ottobre_29/01-politico-unocorriere-web-sezioni-83d2f8c6-195c-11eb-b299-55dcc243e9fe.shtml>. Accessed 13 October 2021.

I7: Sarzanini, Fiorenza. 2020. Miozzo (Cts): “Le scuole chiuse sono la vera emergenza. Dobbiamo riaprirle”. *Corriere della Sera*. 16 November 2020.<https://www.corriere.it/politica/20_novembre_16/miozzo-cts-le-scuole-chiuse-sono-vera-emergenza-dobbiamo-riaprirle-31050d5e-2777-11eb-80dd-837b5190599c.shtml>. Accessed 13 October 2021.

I8: Salvatori, Clarida, 2021. Vaia (Spallanzani): “Il virus colpisce i più deboli. Vaccini? Gli effetti non prima di febbraio”. *Corriere della Sera*. 3 January 2021.<https://roma.corriere.it/notizie/cronaca/21_gennaio_03/vaia-il-virus-attaccacon-piu-forza-poverie-categorie-disagiate-a6846f72-4d31-11eb-b0d1-55bf7888f187.shtml>. Accessed 13 October 2021.

I9: Locatelli, Franco. 2021. Video declarations by Franco Locatelli, at SkyTG24. *TVRep.* 21 December 2020.<https://video.repubblica.it/dossier/coronavirus-wuhan-2020/covid-locatelli-cts-improbabile-che-variante-gb-resista-ai-vaccini/373435/374049>. Accessed 13 October 2021.

I10: Speranza, Roberto. 2020.Video declarations by Roberto Speranza, at “Mezz’ora in più” on Rai 3. *La Repubblica* 4 October 2020. <https://www.repubblica.it/cronaca/2020/10/04/news/speranza_se_dobbiamo_rischiare_facciamolo_per_le_scuole_non_per_gli_stadi-269441205/?ref=search> and<https://www.raiplay.it/video/2020/09/Mezzora-in-piu-bfcde7dd-365e-4ae7-9a11-fa2c821d1833.html>. Accessed 13 October 2021.

I11: Sileri, Pierpaolo, and Luca Richeldi. 2020. Video declarations by Pierpaolo Sileri and Luca Richeldi at Dimartedì. *La7.*  6 October 2020.<https://www.la7.it/dimartedi/rivedila7/dimartedi-puntata-del-06102020-07-10-2020-343371> Accessed 13 October 2021.
